# Let’s Stalk about Membranes: Committor-Based Enhanced Sampling of Stalk Formation

**DOI:** 10.1021/acs.jpclett.6c02454

**Published:** 2026-08-31

**Authors:** Giorgia Rossi, Enrico Trizio, Davide Bochicchio, Giulia Rossi, Michele Parrinello

**Affiliations:** † Atomistic Simulations, 121451 Italian Institute of Technology, 16156 Genova, Italy; ‡ Department of Physics, University of Genova, 16146 Genova, Italy; § INFN Sezione di Genova, 16146 Genova, Italy

## Abstract

Membrane fusion is essential for cellular communication and function, and understanding how two lipid bilayers merge is the key to informing therapeutic strategies. Functionalized nanoparticles have recently emerged as synthetic fusogens, but the molecular mechanisms driving this process remain unclear, partly because fusion involves transitions over high free-energy barriers, which are difficult to capture in molecular simulations. While enhanced sampling methods can address this problem, they also rely on the definition of collective variables, which are especially hard to define for fusion as it arises from the collective rearrangement of many molecules and cannot be easily reduced to a simple intuitive coordinate. Here, we study stalk formation, the first step of fusion, mediated by an amphiphilic gold nanoparticle, by employing an enhanced sampling strategy based on the committor function, machine-learned through a self-consistent procedure. This method requires minimal prior knowledge of the system and leverages the learned committor function as an effective collective variable, enabling the uniform sampling of the entire pathway. From the resulting reactive trajectories and extensive transition region sampling, we obtained converged free-energy estimates and mechanistic insight into stalk formation.

## Graphical abstract

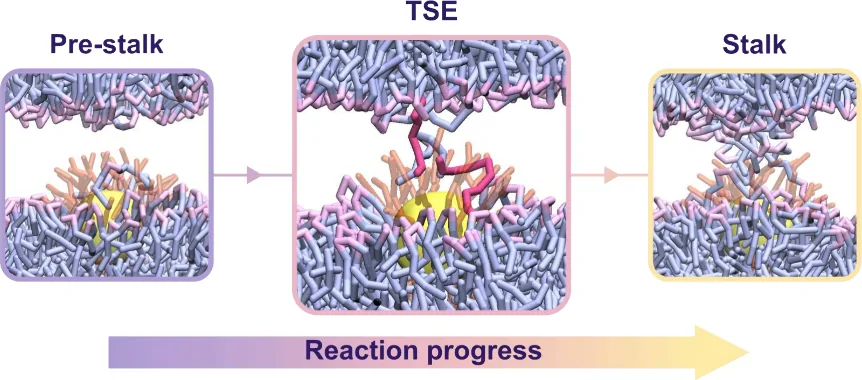

Membrane fusion is the topological transformation by which two lipid bilayers merge, enabling lipid mixing and, ultimately, the exchange of aqueous contents between the two original membrane-bound compartments. Although fusion is central to many cellular processes, from vesicular trafficking to membrane repair and viral entry, it is increasingly important as an engineering principle. Synthetic fusogenic agents, including peptides, nucleic acids, polymers, lipid nanoparticles, and inorganic nanoparticles, are now being developed to trigger fusion in controlled settings for applications in synthetic biology, nanomedicine, and intracellular delivery.^1,2^ In this context, the central question is no longer simply how biological fusion occurs but how molecular design parameters can be tuned to make fusion efficient, selective, and predictable.

Synthetic fusogens exploit different physical routes to favor membrane fusion. They may promote membrane adhesion and dehydration, generate curvature, and perturb local lipid packing, e.g., by stabilizing lipid protrusions. Gold nanoparticles functionalized with mixed hydrophobic and charged ligands provide one representative example: their amphiphilic character enables membrane insertion and interactions with lipid headgroups, making them effective promoters of membrane fusion.^3–7^ However, more generally, synthetic fusogens span a broad design space, where size, shape, surface chemistry, flexibility, and membrane-anchoring geometry may all affect the fusion pathway. Rational design therefore requires a mechanistic understanding of how these molecular features reshape the fusion pathway and alter the stability of its intermediate states.

At the molecular level, fusion between lipid bilayers proceeds through collective rearrangements. Once apposed membranes are brought into close contact (∼2 nm), local dehydration and lipid tail protrusions can trigger the formation of the first hydrophobic bridge between bilayers. This is referred to as the stalk, a key kinetic bottleneck that may evolve toward the fusion pore.^8^ Molecular dynamics simulations can resolve this process at the nanoscale and, in the presence of synthetic fusogens, connect fusogen design features to microscopic mechanisms. However, these transitions occur on time scales that exceed those of standard approaches, as they require crossing large free energy barriers. As a consequence, they are rare events in simulations, thus making their direct observation challenging. Conveniently, several strategies can be devised to alleviate this limitation by making simulations both faster and more efficient. For instance, significant speed-ups can be obtained using a coarse-grained (CG) representation of the system to describe the interatomic interactions that drive the dynamics. By reducing the number of degrees of freedom and smoothing the underlying energy landscape, CG models substantially extend the accessible length and time scales compared to all-atom force fields. However, even with the use of CG models, the observation of spontaneous fusion events remains challenging in simulations.

This calls for the use of enhanced sampling techniques,^9,10^ which add an external bias potential to the original energy landscape to lower effective barriers in order to observe reactive events, reconstruct free-energy landscapes, and characterize the transition state ensemble. In many of these methods, the bias is expressed as a function of a few collective variables (CVs), which are continuous and differentiable functions of the atomic coordinates. Therefore, the effectiveness of these methods strongly relies on the definition of an appropriate CV. If the CV is poorly chosen, it may lead to hysteresis, overlook important slow degrees of freedom, and artificially collapse metastable and transition state configurations into overlapping regions of the projected space. Several enhanced sampling strategies have been applied to membrane fusion, including umbrella sampling^11–14^ and path-finding approaches.^15–17^ These methods, using CVs, such as intermembrane distance, lipid tail connectivity, local density fields, or path coordinates in a reduced CV space, have made it possible to compute free-energy profiles for the formation of early fusion intermediates and to clarify how lipid composition, hydration, and membrane stress affect the fusion barrier.^18^

In this work, we adopt a different strategy based on the committor function *q*(**x**), which, given two metastable states A and B, returns the probability that a trajectory initiated from configuration **x** reaches state B before passing through A.^19^ As a consequence, it provides a rigorous probabilistic description of the reaction progress and is considered to be the optimal reaction coordinate. However, determining the committor is not trivial. Recently, we have proposed a self-consistent iterative procedure in which machine learning and enhanced sampling are combined to leverage the variational principle the committor obeys.^20,21^ Besides learning a parametrization for the committor, this method allows the rare event to be characterized in detail, starting from information limited to the initial and final states of the process. To this end, the committor is represented as a neural network *q*(**d**(**x**)) that takes as input a set of physically motivated descriptors *d*(**x**) and is optimized via a loss function encoding the variational principle. This, in practice, amounts to minimizing the expectation value of the gradients of the committor with respect to the atomic positions **x** while enforcing appropriate boundary conditions. A progressively improved estimate of the committor is obtained through an iterative, self-consistent procedure, in which we alternate between sampling and learning. At each iteration, the current estimate of the committor is used both as a CV to drive enhanced sampling simulations and to construct a bias potential aimed at increasing the sampling of the otherwise unfavored transition state region. The configurations thus sampled are then used as the training dataset for the next iteration step, and the cycle is iterated until convergence.

The original formulation of this approach has been successfully applied to a wide range of systems,^20–24^ but when the relevant degrees of freedom involve a large number of atoms, as is the case for membrane fusion, it can become too computationally demanding due to the explicit calculation of the gradients with respect to the atomic coordinates. To overcome this limitation, we recently introduced^25^ an approximated variational functional that bypasses the expensive calculation of coordinate gradients and simply uses the much cheaper gradients with respect to the input descriptors. Although this approximated principle does not formally target the exact committor function, it still retains the ability to enhance sampling and to characterize the TS ensemble, thus providing an efficient tool for the study of complex processes, such as membrane fusion, where both the dimensionality of the system and the complexity of the relevant degrees of freedom pose significant challenges.

In this work, we apply this committor-based enhanced sampling framework to the study of stalk formation induced by a functionalized amphiphilic gold nanoparticle. The input descriptors that we chose are indicators of the presence and quality of a hydrophobic connection between the bilayers and are general enough to be transferable to the study of fusion induced by other synthetic or biogenic fusogens. We demonstrate that this approach provides an effective and semi-automatic tool to study stalk formation and is able to characterize the complex dynamics of the process by uniformly sampling the whole reactive pathway, observing many reactive events, and extensively sampling the transition state region. As a result, we obtained accurate free energy profiles and performed an in-depth probability-based analysis of the different stages leading to stalk formation. Being semi-automatic, fast, and easy to set up, this method provides a flexible, general tool for studying fusion processes.

## Method: Committor Function

Given two metastable states A and B, the committor function *q*(**x**) is defined as the probability that a trajectory initiated from configuration **x** reaches state B before visiting A. By construction, it satisfies the boundary conditions *q*(**x**_*i*∈A_) = 0 and *q*(**x**_*i*∈B_) = 1 and varies smoothly between these values across the transition state (TS) region. Owing to its probabilistic nature, the committor provides a rigorous description of the progress of a rare event and is widely regarded as the optimal reaction coordinate. However, its direct determination is challenging. One possible route to obtain it is to use a variational principle, which, under the assumption of overdamped Langevin dynamics, states that the committor can be obtained by imposing the boundary conditions above and minimizing the functional $K\left[q\left(x\right)\right]=\left\langle {\left|\nabla_{u}q\left(x\right)\right|}^{2}\right\rangle$ , where ∇_**u**_ denotes the gradient with respect to mass-weighted coordinates and ⟨·⟩ is the Boltzmann average.

## Method: Machine Learning the Committor

Following ref 20, we introduce a machine-learning parametrization of the committor, which is progressively refined through alternating cycles of training and sampling. In this framework, the committor is represented as a feed-forward neural network (NN) *q*_**θ**_(**x**) = *f*_**θ**_(*d*(**x**)), parametrized by a set of learnable weights **θ** and taking as input a set of physical descriptors *d*(**x**). The parameters **θ** are optimized by minimizing a loss function *L* = *L*_v_ + α*L*_b_, composed of two terms scaled to have the same order of magnitude by the hyperparameter α. The boundary loss *L*_b_ enforces the correct boundary conditions [i.e., *q*(**x**_*i*∈A_) = 0 and *q*(**x**_*i*∈B_) = 1] and is evaluated on a labeled dataset of configurations collected from short unbiased simulations in the metastable basins and labeled accordingly. The variational loss *L*_v_ encodes the variational principle for the committor discussed above and is evaluated on configurations with the associated statistical weights. In its original formulation, *L*_v_ requires computing the gradients of the committor with respect to the mass-scaled atomic positions **u**, possibly making its evaluation computationally expensive. This cost becomes particularly significant when the descriptors involve complex functions of atomic coordinates or depend on a large number of atoms, as in membrane fusion. This introduces a critical bottleneck in training, making the method unfeasible for such systems. To alleviate this limitation, we adopt the reformulation that we recently introduced in ref 25, in which the coordinate gradients present in the original variational functional are restricted to the descriptor space (i.e., ∇_**u**_ → ∇_**d**_), thus leading to the approximated and much easier to compute variational functional

1 $$\mathrm{K̃}\left[q\left(d\left(x\right)\right)\right]=\left\langle {\left|\nabla_{d}q\left(d\left(x\right)\right)\right|}^{4}\right\rangle$$

which can be shown to provide an upper bound to the original functional and is used to define the new variational loss in the training procedure. Although this new approach does not formally target the exact committor function, it has been shown that minimizing the modified functional in eq 1 yields a committor model that remains effective in driving efficient enhanced sampling simulations. As a result, this method provides a reliable and computationally efficient alternative, enabling a substantial reduction in training cost for complex systems.

## Method: Enhanced Sampling Scheme

Effective training of the committor model *q*_**θ**_(**x**) requires a data set containing configurations over the whole relevant phase space, including both the metastable basins and the TS region. To this end, two complementary bias potentials are applied simultaneously, as proposed in ref 21. The first is the transition-state-oriented Kolmogorov bias introduced in ref 20

2 $$V_{K}\left(x\right)=-\frac{\lambda }{\beta }\log\left(|\nabla_{d}q\left(x\right){|}^{2}+\epsilon \right)$$

where $\beta ={\left(k_{B}T\right)}^{-1}$ is the inverse temperature, λ controls the strength of the bias, ϵ is a regularization parameter, and the gradients ∇_**d**_ *q*(**x**) are calculated with respect to the descriptors, as proposed in ref 20. The bias *V*_*K*_(**x**) is attractive in regions where |∇*q*(**x**)| is large, i.e., in the TS region, and therefore enhances its sampling. The λ parameter is tuned to yield a bias potential *V*_*K*_(**x**) with a magnitude comparable to that of the expected free energy barrier, resulting in efficient sampling of the TS region.

However, *V*_*K*_(**x**) alone does not ensure an efficient exploration of the metastable states. For this reason, it is combined with the metadynamics-like on-the-fly probability enhanced sampling (OPES) method,^26–28^ which works by progressively building a repulsive bias that discourages the sampling of already visited configurations. The OPES bias is defined in the space of a chosen collective variable (CV, **s**), which is a function of the atomic coordinates, i.e., **s** = **s**(**x**). In practice, the OPES bias is built so as to drive sampling toward a target distribution *p* ^tg^(**s**), in which the probability of observing reactive events is enhanced. To achieve this, OPES iteratively builds an estimate of the system’s probability distribution along the CV and constructs a bias potential that drives the sampled distribution toward *p* ^tg^(**s**). In practice, we apply the OPES explore^27^ bias *V*_OPES_(**x**) on the committor-related variable *z*(**x**) introduced in ref 21, which is related to the committor via q(x)=σ(z(x)) , where σ is a sigmoid-like activation function. This change of variable is performed because the sharp behavior of *q*(**x**) makes it unsuitable as a CV for enhanced sampling, at variance with *z*(**x**), which encodes the same physical information in a smoother form.

The combined action of *V*_*K*_(**x**) and *V*_OPES_(**x**) results in a sampling scheme that uniformly covers the full reactive process, simultaneously promoting transitions between metastable states and increasing the sampling of the crucial transition state region.

## Method: Self-Consistent Iterative Procedure

The neural network parametrization and the enhanced sampling scheme are combined into the self-consistent iterative procedure summarized here.^20,21^

- Step 1: At iteration *n*, the committor model $q_{\theta }^{n}\left(x\right)$ is trained on the dataset of configurations **x** ^*n*^ and weights **w** ^*n*^ available at that point. At the first iteration (*n* = 0), the model is trained only on the labeled configurations, obtained by running short unbiased simulations in the metastable basins.
- Step 2: Enhanced sampling simulations are performed under the combined action of $V_{\mathrm{OPES}}^{n}\left(x\right)$ , applied on the committor-related CV *z*(**x**), and the Kolmogorov bias $V_{K}^{n}\left(x\right)$.
- Step 3: The configurations sampled at step 2 are treated as the new training set, after being reweighted according to the effective bias $V_{\mathrm{eff}}^{n}\left(x\right)=V_{K}^{n}\left(x\right)+V_{\mathrm{OPES}}^{n}\left(x\right)$ with weights given by $w_{i}^{n}=e^{\beta V_{\mathrm{eff}}^{n}\left(x_{i}\right)}/{\left\langle e^{\beta V_{\mathrm{eff}}^{n}\left(x\right)}\right\rangle }_{U_{\mathrm{eff}}^{n}}$ .

The above steps are repeated iteratively to progressively refine both the committor estimate and the sampling, until convergence is achieved.

## Results

As anticipated, we investigate stalk formation in water between two parallel (25 × 25) nm membrane patches, both composed of 1,2-dioleoyl-*sn*-glycero-3-phosphocholine (DOPC) lipids. The process is mediated by an Au nanoparticle (AuNP) with a core diameter of 2 nm, functionalized with 11-mercapto-1-undecanesulfonate (MUS) and octanethiol (OT) ligands, and embedded in the lower bilayer. The functionalized AuNP, the lipids, and the solvent were modeled at the coarse-grained level with the Martini 2 ^29^ force field, as detailed in our previous works.^5,30^ The system was hydrated with a water-to-lipid ratio of about 12 Martini water beads per lipid molecule and maintained at a temperature of 310 K.

Preliminary simulations reveal that, at the level of dehydration considered here, before the stalk is formed, the membranes can adopt two distinct configurations. In the pre-stalk state (state A), the membrane is locally curved and the NP ligands remain in contact with the upper bilayer, maintaining the two membranes in close apposition. In a secondary state A′, instead, the ligands detach from the upper leaflet, and the membranes relax toward a flatter configuration. Working under moderate rather than extreme dehydration is desirable because it allows the membranes to retain more natural shape and thickness fluctuations and is therefore expected to provide a more realistic description of the local rearrangements leading to stalk formation. At the same time, these fluctuations make it possible for the system to leave the pre-stalk state by membrane detachment. Since our aim is to sample the transition from the pre-stalk state A to the stalk state B rather than the reversible association and dissociation of the two membranes A ↔ A′, we prevent visits to the detached A′ state by applying a lower wall restraint on the number of contacts between the terminal ligand beads and the lipid headgroups of the upper membrane.

Because NP-mediated stalk formation is localized in the intermembrane region above the embedded NP, we designed a set of input descriptors for the committor model to account for the presence of hydrophobic tails in this region. As stalk formation is initiated by the accumulation and reorientation of lipid tails between the two bilayers, we define the descriptors as the number of lipid tail beads contained in 17 concentric spherical shells centered on the NP and restricted to the intermembrane region (Figure 1). This can be seen as a generalization of the geometric construction of the chain coordinate,^13,14,31^ without the imposition of a pre-defined directionality to the orientation of the stalk structure. In addition, we apply two geometric restrictions to the shells to consider in the calculations only the tails that actively contribute to stalk formation: on one hand, a height cutoff that excludes lipids below the surface of the NP and, on the other hand, a lateral cutoff of 2 nm from the NP center, to avoid the application of bias to regions too far from the NP. Together, this localizes the descriptors to the region where the hydrophobic bridge forms. Conveniently, this overall construction also allows the use of a single shared neighbor list, which minimizes the computational overhead associated with descriptor evaluation.

**Figure 1:**
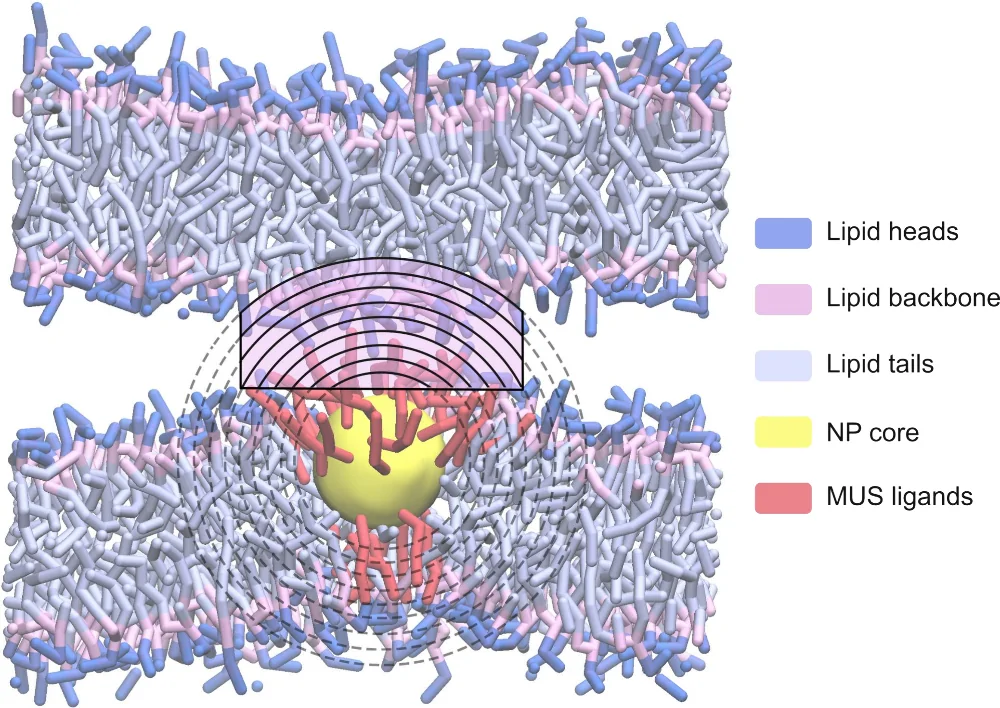
Descriptor construction. Cross-section of two apposed DOPC bilayers with a NP embedded in the lower membrane. Lipid tail coordination numbers are computed within concentric spherical shells centered on the NP center of mass. Only the shaded region is retained in the descriptor definition: a height cutoff excludes lipid tails below the NP surface, while a lateral cutoff confines the analysis to the intermembrane region directly above the NP, where stalk formation occurs.

Once the descriptor set was chosen, we trained a committor model *q*_**θ**_ using the self-consistent iterative protocol of ref 25, which we fully detail in the Methods. A test of the robustness of the committor against alternative choices of the descriptors in terms of the number and thickness of shells is reported in Figure S2.

Enhanced sampling is driven by two complementary bias potentials. On one hand, the Kolmogorov bias *V*_*K*_(**x**) enhances the sampling of the transition state ensemble, and on the other hand, the explore variant of the metadynamics-like OPES bias^26,27^ progressively fills the free energy landscape along the committor-related CV *z*(**x**), promoting transitions between the pre-stalk and stalk basins.

At convergence, which was reached after two iterations, we used the committor model thus obtained to perform a set of five independent 1 μs production simulations to gather meaningful statistics. The free energy estimates obtained via reweighting from these calculations are reported in Figure 2. These are compatible with results obtained using the chain coordinate^5^ (see the Supporting Information) and show a consistent behavior across all replicas, thus confirming the reliability of our calculations.

**Figure 2:**
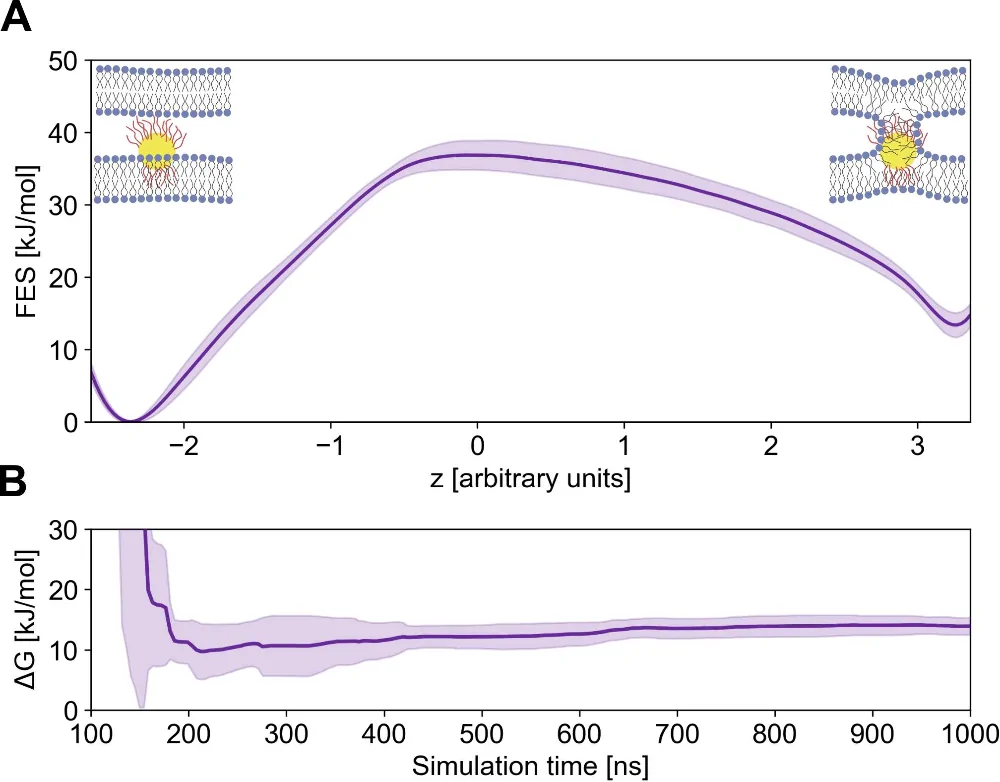
Free energy estimates. (A) Free energy surface (FES) for the stalk formation projected on the committor-related CV *z*(**x**). The insets schematically depict the two states and are placed above the corresponding CV values. (B) Estimate of the free energy difference Δ*G* between the two states as a function of the simulation time. In both panels, the solid line reports the average over five independent 1 μs replicas, while shaded regions indicate the associated statistical errors. In both cases, an initial transient of 100 ns has been discarded.

Beyond free energy reconstruction, the learned committor provides insight into the reaction mechanism, since the variable *z*(**x**) naturally orders the reaction progress.^20,21^ We thus grouped the sampled configurations into five bins according to their *z*(**x**) values, ordered from the no stalk basin to the stalk basin. For each bin, we identified the most representative structure using *k*-medoids clustering^32^ in a space combining the lipid tail descriptors with an equivalent set of water coordination variables, defined using the same shell geometry (Figure 3A). Inspection of these representative structures reveals the gradual rearrangement of the lipids during the transition. In the transition state bin, the hydrophobic connection involves only a few lipids: one lipid from the lower membrane protrudes upward, while a lipid from the upper membrane exposes one tail toward the intermembrane region together with a partially detached lipid located between the two leaflets. The transition state ensemble therefore contains only a small, localized hydrophobic connection, marking the onset of interleaflet contact before the recruitment of additional lipids produces a fully developed stalk. In the fourth bin, the connection broadens through the recruitment of lipids from both membranes before evolving into the more compact and continuous hydrophobic structure observed in the stalk basin. The progression also highlights the different roles of the two membranes. The lower membrane undergoes strong local fluctuations, with repeated lipid tail protrusions toward the intermembrane space as a consequence of the NP-induced perturbation. These fluctuations alone, however, are not sufficient to initiate the stalk formation. The transition progresses only when a lipid from the upper membrane reorients one of its tails toward this region, establishing the first hydrophobic contact between the two leaflets (see Figure S6). It is worth noting that this analysis is made possible by the uniform OPES + *V*_*K*_(**x**) sampling, which provides a large number of configurations across the entire transition pathway, including the TS region, retaining sufficient statistics in each bin.

**Figure 3:**
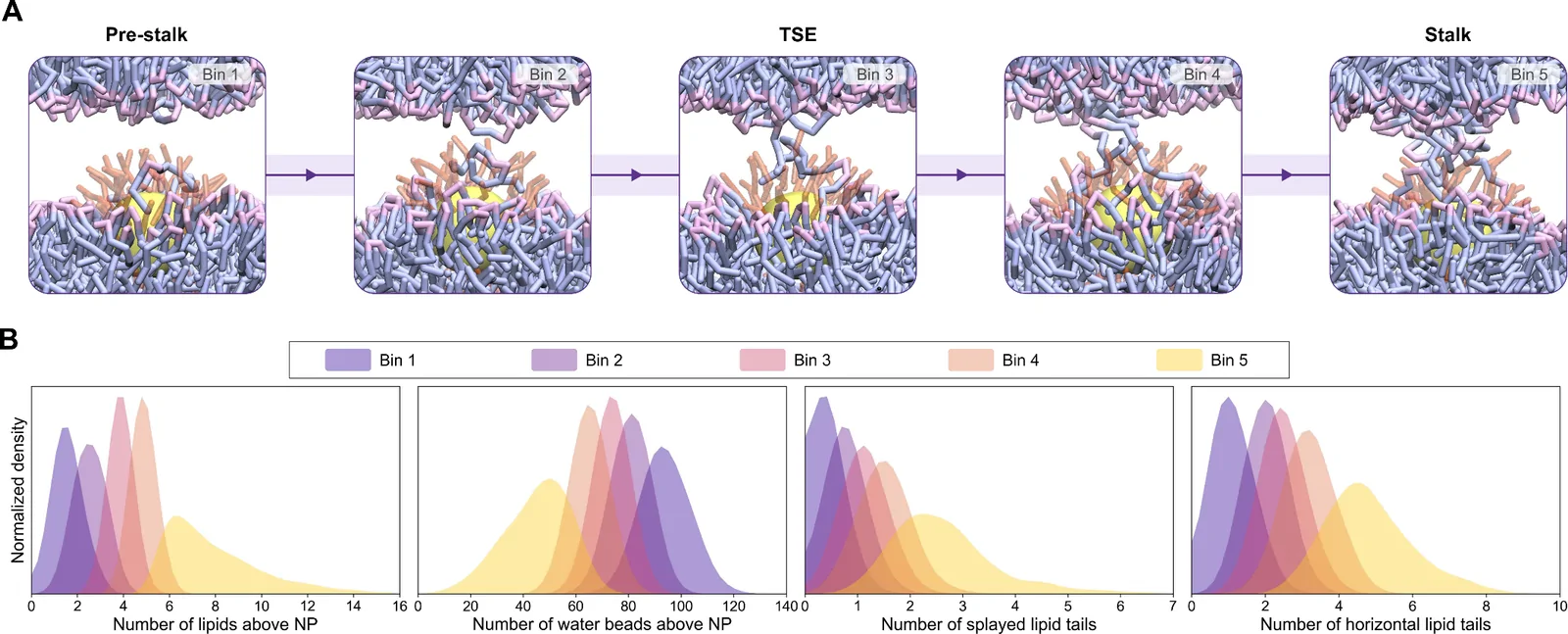
(A) Representative medoid configurations from five bins ordered according to their committor value, showing the progressive formation of the stalk between the two bilayers. (B) Normalized distributions of four structural descriptors across the five committor bins, respectively: number of lipids above the NP, number of water beads above the NP, number of splayed lipid tails, and number of horizontally oriented tail segments. The configurations were assigned to bins using *z* obtained from iteration 3 of the committor model. The intervals are bin 1, −2.2 ≤ *z* < −1.5; bin 2, −1.5 ≤ *z* < −0.5; bin 3, −0.5 ≤ *z* < 0.5; bin 4, 0.5 ≤ *z* < 1.5; and bin 5, 1.5 ≤ *z* ≤ 2.7.

Beyond the qualitative information that single structures provide, we also characterize the process quantitatively by computing ensemble averages of several quantities relevant to stalk formation dynamics, which report on the local hydration and lipid rearrangement above the NP (Figure 3B). Moving from the pre-stalk basin to the stalk basin, the number of lipid tails above the NP increases, while the number of water beads above the NP decreases. In addition, the transition is accompanied by the presence of an increasing number of splayed lipid tails and horizontally oriented tails, consistent with the collective lipid reorientation and protrusion events that precede the hydrophobic stalk formation. Together, these trends show how stalk formation proceeds through the combined expulsion of water, recruitment of lipid tails from both leaflets, and their progressive reorientation into a continuous hydrophobic connection, which underlies the collective nature of the transition.

## Discussion

In this work, we applied our recently developed committor-based enhanced sampling framework to study NP-mediated stalk formation. Starting from information limited to the pre-stalk and stalk states only, the committor estimate was progressively refined through alternating cycles of learning and enhanced sampling, and the resulting committor-related variable was used to promote repeated transitions across the full pathway while enhancing the sampling of the transition state region. This resulted in a uniform exploration of the reactive process, converged free energy estimates, and mechanistic insight into the transition mechanism.

As with any other machine-learning-based method, the effectiveness of our approach depends on the choice of the descriptors used as inputs to the model. In this regard, the concentric lipid tail coordination shells adopted here, which preserve the physical information on hydrophobic connectivity CVs, represent a flexible option that can be applied to describe fusion processes mediated not only by NPs but also by other fusogenic peptides, proteins, and synthetic molecules or, even in the absence of fusogenic agents, possibly adjusting the location of the spheres relative to the stalk position. In addition, our approach is also robust and not too sensitive with respect to the choice of the shell geometry, as proven by the agreement between models trained with different shell numbers and thicknesses, and the descriptors are also computationally efficient as all shells share a common neighbor list.

Even if for the studied system these descriptors proved informative enough to obtain good sampling and converged free energy estimates, thus representing a promising option also for more complex systems, we note that, in such cases, additional descriptors could also be included to better capture finer and distinct degrees of freedom to further accelerate the dynamics. For example, descriptors related to membrane curvature, lipid orientation, or specific fusogen–lipid interactions could be incorporated to study mechanisms in which the transition mechanism is more strongly related to membrane deformation or molecular recognition. It is also worth noting that the vertical and lateral cutoffs used here deliberately focus the descriptors on the region above the NP, where stalk formation is expected to occur. While this introduces reasonable and somewhat general prior information about the location and direction of the stalk during the process, these restrictions could be relaxed and adapted to different fusogen geometries to provide a less directional representation in which the position and orientation of the hydrophobic connection emerge directly from the simulations.

Beyond its sampling efficiency and flexibility, the probabilistic description provided by the committor is particularly well-suited to describe the complexity of membrane fusion, in which the reaction progress emerges from several coupled and collective molecular rearrangements rather than from a single event. Ordering configurations along the committor shows that the transition proceeds when lipid tails from the upper membrane reorient toward the hydrophobic region above the NP and establish the first hydrophobic contact. This is accompanied by gradual water depletion and recruitment, splaying, and reorientation of multiple lipids, supporting a cooperative mechanism of stalk formation.

Overall, this work shows a general, semi-automatic, and efficient framework for the study of stalk formation, which, starting from very limited information, leads to deep insights into fusion mechanisms and thermodynamics. We believe that this, combined with the flexibility of the whole procedure, will provide a precious tool to better understand fusion processes under realistic conditions.

## Supplementary Material



## Data Availability

The committor models were trained using the open-source library mlcolvar.^33^ Molecular dynamics simulations were performed using GROMACS,^34^ with a custom interface for the PLUMED^35,36^ plugin for enhanced sampling and free-energy calculations. Simulation inputs and the code needed for training and performing enhanced sampling simulations are available in the GitHub repository https://github.com/giorgiarossii/membrane_fusion_committor.git. Input files of the enhanced sampling simulations are also available on the PLUMED-NEST repository with plumID: 26.011.

## Notes

Complete computational and committor model training details, all molecular dynamics simulations carried out using GROMACS-2024.5 patched with PLUMED-2.9.4 and the Martini 2 force field, implementation and PLUMED input examples for the multi-radius coordination descriptors and the adapted chain coordinate, analyses of committor model convergence and descriptor sensitivity, comparison of free-energy estimates and sampling obtained with the committor-based and chain coordinate approaches, and analysis of hydrophobic tail ligand contacts during stalk formation (PDF)
