# Supplementary material for: Let’s Stalk about Membranes: Committor-Based Enhanced Sampling of Stalk Formation
Source: J Phys Chem Lett. 2026 Aug 31;17(36):10518–24. doi: 10.1021/acs.jpclett.6c02454 (PMC13573609; doi:10.1021/acs.jpclett.6c02454)
Supplement: Supplementary file 1 [file jz6c02454_si_001.pdf]

Let's Stalk About Membranes: Committor-Based Enhanced Sampling of Stalk Formation

Supporting Information

Giorgia Rossi,[†,‡] Enrico Trizio,[†] Davide Bochicchio,[‡,¶] Giulia Rossi,[‡,¶] and Michele Parrinello[*,†]

†*Atomistic Simulations, Italian Institute of Technology, 16156 Genova, Italy*

‡*Department of Physics, University of Genova, 16146 Genova, Italy*

¶*INFN sezione di Genova, 16146 Genova, Italy*

E-mail: michele.parrinello@iit.it

Computational details

Simulation details All simulations were carried out targeting the NPT ensemble, using the GROMACS-2024.5[1] MD engine patched with PLUMED-2.9.4[2,3] and the Martini 2[4] force field.

Two apposed DOPC bilayers separated by an initial distance of 3 nm, in a (25 × 25 × 15) nm simulation box were simulated in the isothermal–isobaric (NPT) ensemble using a time step of 20 fs. Temperature was maintained at 310 K using the velocity-rescale thermostat[5] with a coupling time constant of 1.0 ps. Pressure was set at 1 bar using the Parrinello–Rahman

barostat with semi-isotropic pressure coupling, and a coupling time constant of 12 ps. Periodic boundary conditions were applied in all three spatial directions.

Committor model training details To model the approximated committor, we used a set of 17 coordination numbers as inputs of a neural network (NN) with architecture [17, 14, 12, 1] nodes/layer and an initial normalization layer. Starting from the standard `COORDINATION` action in PLUMED, we implemented a custom multi-radius coordination function, `COORDINATION_MULTI`. This action computes smooth coordination numbers between the NP center of mass and the lipid tail beads for multiple radii in a single call.

```
# Define the nanoparticle group from the index file
NP: GROUP NDX_FILE=index.ndx NDX_GROUP=NP
# Define the lipid tail bead group from the index file
tails: GROUP NDX_FILE=index.ndx NDX_GROUP=tails
# Compute the nanoparticle center of mass
com: CENTER ATOMS=NP
# Compute multi-radius coordination numbers between NP COM and lipid tails
c_tails: COORDINATION_MULTI GROUPA=com GROUPB=tails D_0=0.0R_0
=1.6,1.7,1.8,1.9,2.0,2.1,2.2,2.3,2.4,2.5,2.6,2.7,2.8,2.9,3.0,3.1,3.2,3.3
NN=50 MM=100 D_MAX=3.6 NLIST NL_CUTOFF=3.8 NL_STRIDE=10 N_OUT=18 Z_SHIFT=1
 XY_RADIUS=2

```

Listing 1: Example of PLUMED input file for `COORDINATION_MULTI` action

Here, `GROUPA=com` defines the NP center of mass and `GROUPB=tails` the target lipid tail beads, the values in `R_0` define concentric spheres radii around the NP, and `N_OUT=18` sets the number of output coordination numbers, one per shell. For efficiency, the neighbor list is built using the largest cutoff radius and then reused for all smaller radii. Shell-specific contributions are then obtained separately in the PLUMED input file by subtracting the coordination values computed for consecutive radii. The calculation is restricted to the region above the NP by `Z_SHIFT=1`, which excludes beads below a plane 1 nm above the NP COM, and by `XY_RADIUS=2`, which retains only beads within 2 nm laterally from the

NP center. The complete implementation is provided in the code availability section. All keywords not explicitly discussed here correspond to the standard parameters of the PLUMED `COORDINATION` action.

OPES+V_K simulations biasing the committor-based z variable, with OPES Explore `PACE` = 500, were performed. During all production simulations, a lower wall was applied to the coordination number between the terminal ligand beads, `MUS4`, and the lipid head beads. This restraint was used to maintain a minimum number of ligand-upper membrane contacts and prevent the system from entering a third substate in which the membranes flatten, and the ligands lose contact with the upper membrane.

For the committor training, from the first iteration on, we discarded the first part of the simulations in which the bias was stabilizing. For the optimization, we used the ADAM optimizer with an initial learning rate of 10^{-3} modulated by an exponential decay with multiplicative factor $\gamma = 0.99995$. The training was performed for 10000 epochs during the first iteration, for 5000 during the second one, and for 40000 epochs during the final one. The α hyperparameter in the loss function was set to 10 for the first iteration and to $10^{-}3$ for the other two, while the γ hyperparameter was set to 1 for the first iteration and to 10^3 for the other two iterations. We optimized the log of the total loss for numerical stability. For each iteration, Table S1 reports the size of the training dataset, the simulation time t_d from which this dataset was collected, the OPES Explore `BARRIER`, the value of λ, and the production simulation time t_s performed with the trained model. In this case, only data from the previous iteration was used for the training.

Additional figures

Table S1: Summary of the iterative procedure.

Iteration	Dataset size	t_d [ns]	OPES `BARRIER` [kJ/mol]	λ	t_s [ns]
0	10000	2*1000	30	30	5*500
1	59000	5*500	30	30	5*500
2	97500	5*500	30	50	5*1000

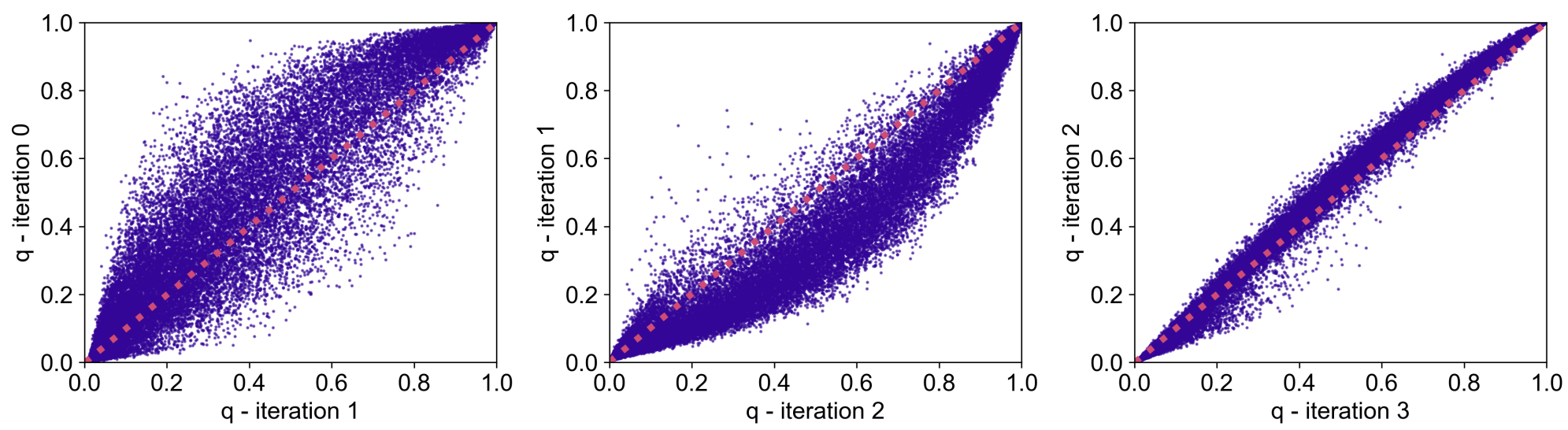

Figure S1: **Convergence of the committor model** across consecutive training iterations. Each panel compares the committor values predicted by two successive iterations, with the dashed red line indicating perfect agreement. The scatter progressively collapses around the diagonal, showing that the model converges over successive iterations. The committor obtained at iteration 2 was used to drive production simulations, while the model was subsequently retrained to verify convergence and to assign configurations to committor-based bins.

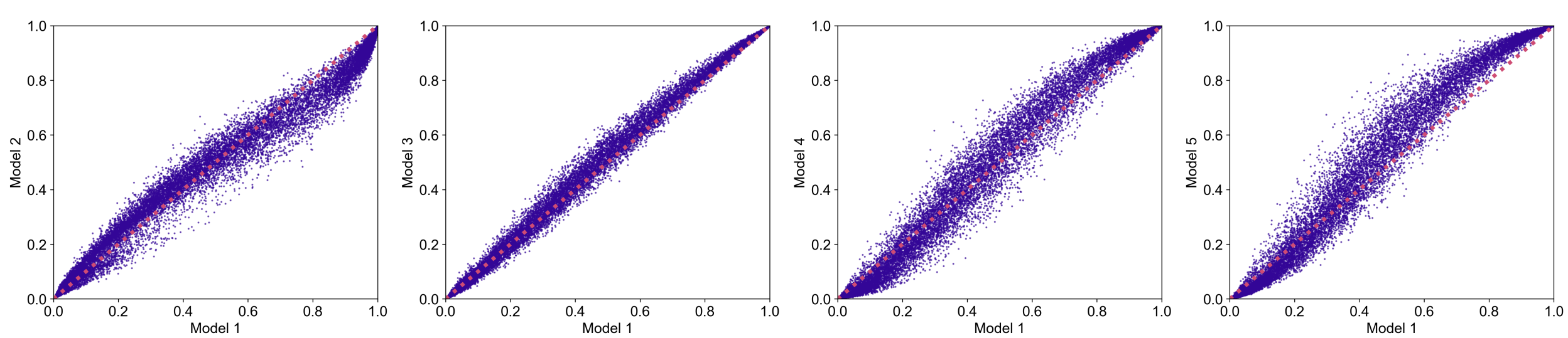

Figure S2: **Agreement between committor models trained with different descriptor sets.** Model 1 is the reference model used in production simulations and was trained with the selected descriptor setup of 17 lipid tail coordination shells with a width of 0.1 nm. It is compared against alternative models trained with 34 shells of 0.05 nm (model 2), 12 shells of 0.15 nm (model 3), 8 shells of 0.2 nm (model 4), and 6 shells of 0.3 nm (model 5), respectively. The x-axis reports the committor predicted by model 1, while the y-axis reports the committor predicted by each alternative model. The dashed red line represents perfect agreement. The overall alignment of the points with the diagonal indicates that the committor prediction is not overly sensitive to reasonable changes in the descriptor sets.

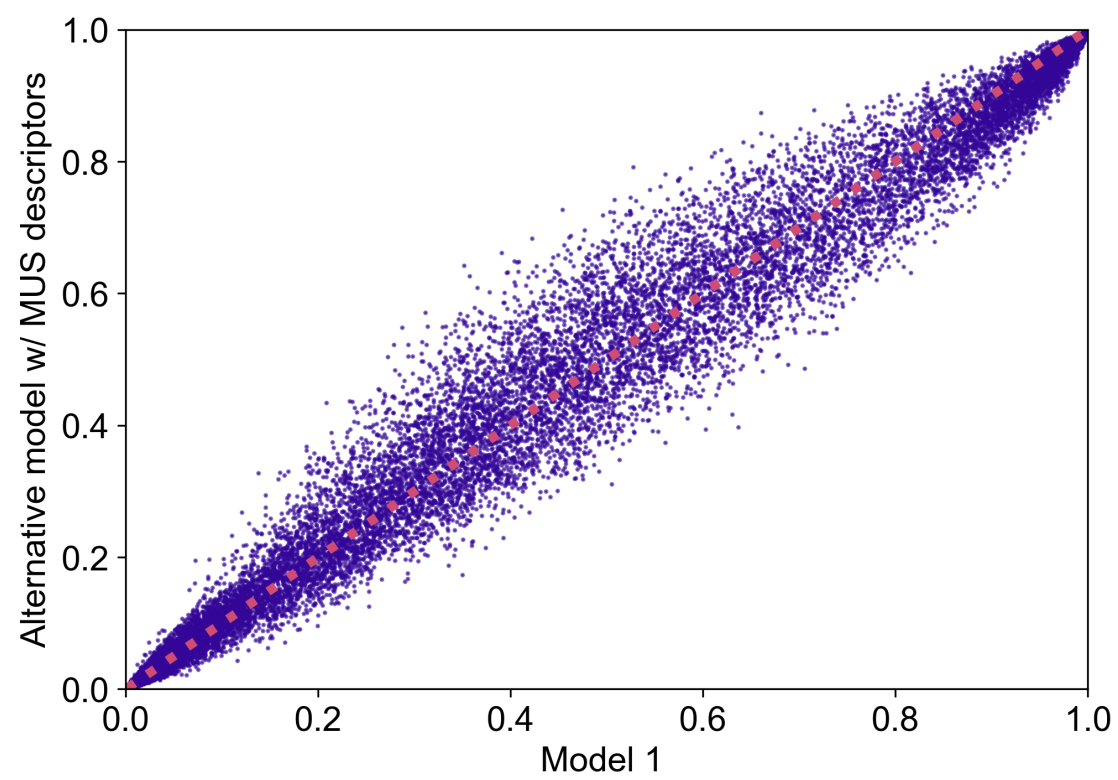

Figure S3: **Agreement between committor models trained with different descriptor sets.** Model 1 is the reference model used in production simulations and was trained with the selected descriptor setup of 17 lipid tail coordination shells with a width of 0.1 nm. This model is compared with an alternative model trained using the same 17 lipid-tail descriptors, supplemented by 6 MUS ligand coordination shells with a width of 0.2 nm, and a lateral cutoff of 1nm with respect to the NP core. The x-axis reports the committor predicted by model 1, while the y-axis reports the committor predicted by the alternative model. The dashed red line represents perfect agreement. The overall alignment of the points with the diagonal indicates that the committor prediction is not overly sensitive to reasonable changes in the descriptor sets.

Chain coordinate

In this work, we used a re-adapted version of the chain coordinate originally introduced by Hub and Awasthi[6] and implemented in PLUMED by Di Bartolo and Masone.[7] Here, the CV was modified to describe NP-mediated stalk formation through the custom PLUMED action `MEMFUSIONP_NP`, available in the code repository. The action was used in the simulations with the following PLUMED input.

```
# Define the nanoparticle group from the index file
NP: GROUP NDX_FILE=index.ndx NDX_GROUP=NP
# Define the lipid tail bead group from the index file
tails: GROUP NDX_FILE=index.ndx NDX_GROUP=tails
# Compute the nanoparticle center of mass
com: CENTER ATOMS=NP
 # Compute the chain coordinate
 memFusion: MEMFUSIONP_NP NP=com TAILS=tails NSMEM=20 DSMEM=0.1 ZSHIFT=1.1 RCYLMEM=2.0

```

Listing 2: Example of PLUMED input file for `MEMFUSIONP_NP` action

Here, `NP=com` defines the NP center of mass, while `TAILS=tails` specifies the lipid tail beads used to evaluate the coordinate. The parameters `NSMEM=20` and `DSMEM=0.1` define 20 slices separated by 0.1 nm along the membrane normal, corresponding to a total sampling height of 2.0 nm. Compared with the original implementation, the cylindrical region is anchored to the NP center of mass instead of being defined from membrane reference groups. Its vertical position is controlled by the new parameter `ZSHIFT=1.1`, which places the cylinder above the NP, while `RCYLMEM=2.0` restricts the calculation laterally to a cylindrical region of radius 2.0 nm around the NP center.

We performed 5 × 1 μs OPES Explore replicas using this adapted chain coordinate as CV, with a `BARRIER` parameter of 30 kJ mol^{-1}.

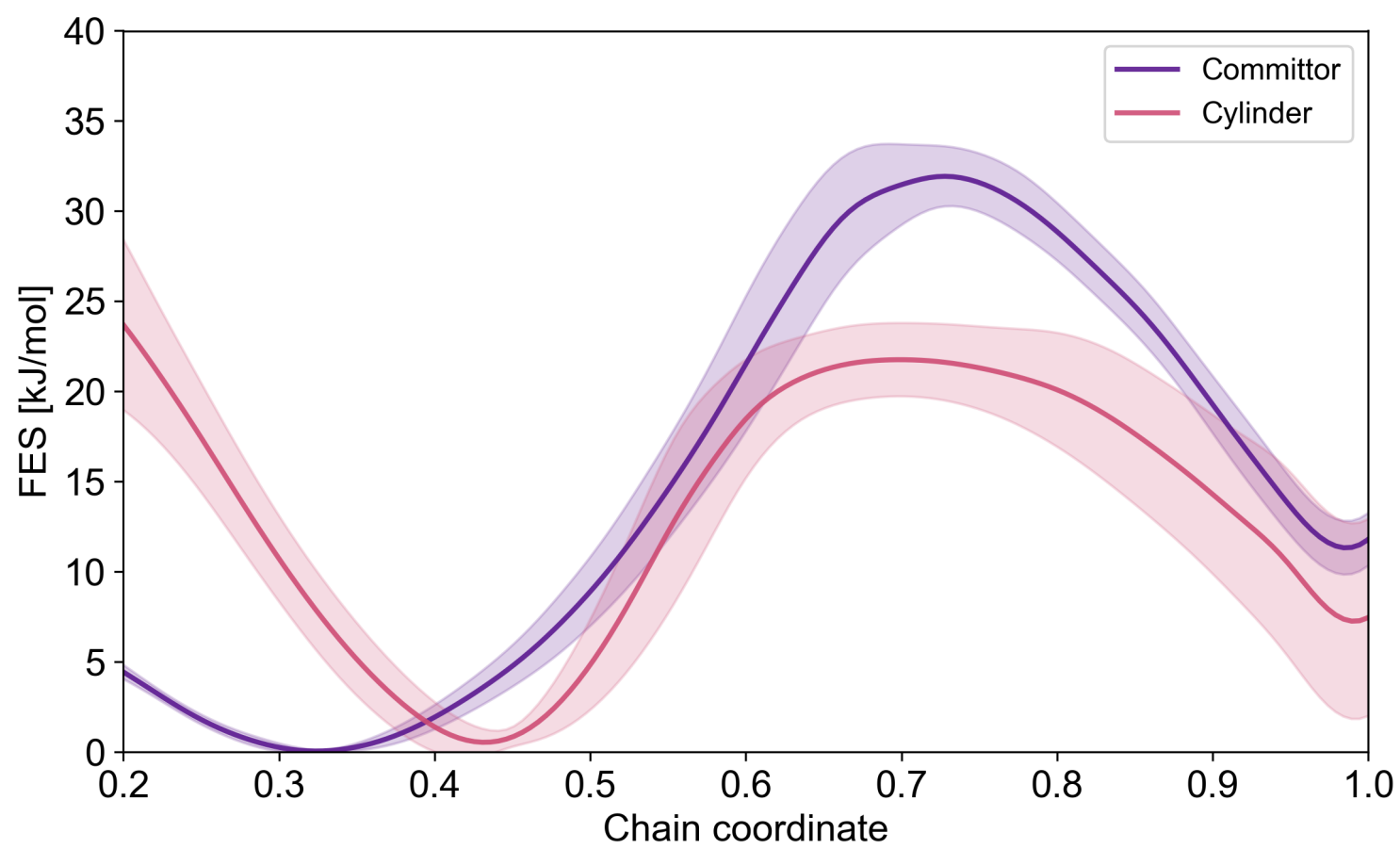

Figure S4: **Comparison between committor and chain-coordinate free energy estimates.** Free energy surface (FES) for stalk formation. For comparison they are both projected on the chain coordinate CV. The committor-based simulations are shown in purple, while the chain-coordinate simulations are shown in pink. Solid lines report the average over five independent 1µs replicas, and shaded regions indicate the associated statistical errors. An initial transient of 100 ns was discarded.

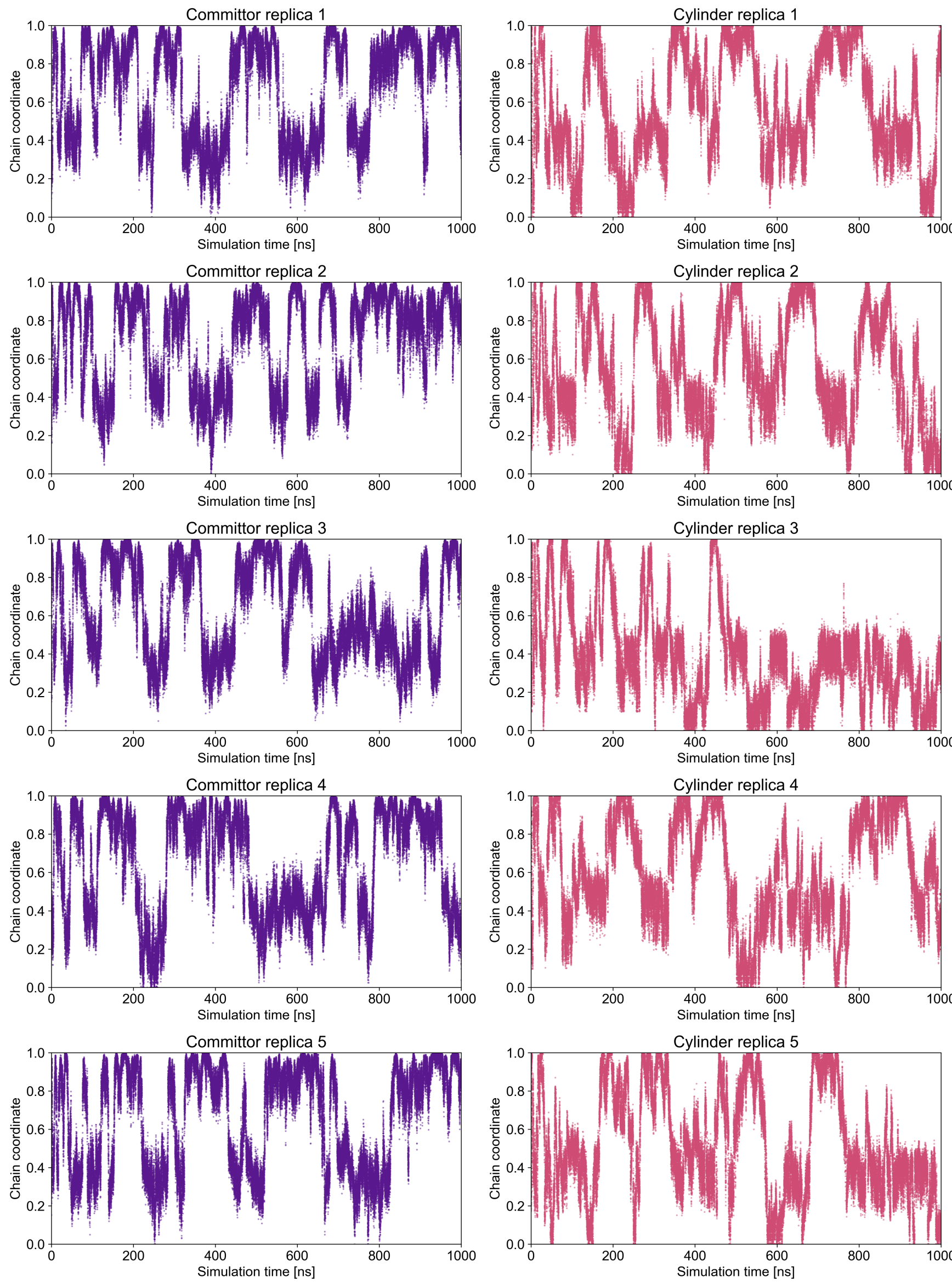

Figure S5: **Comparison of sampling obtained with committor-based and chain-coordinate approaches.** Time series of the chain coordinate for five independent 1 μs simulations performed using the committor-based framework (left, purple) and using the chain coordinate (right, pink). For reference, no-stalk configurations correspond to chain coordinate values < 0.5, whereas stalk states correspond to values > 0.8.

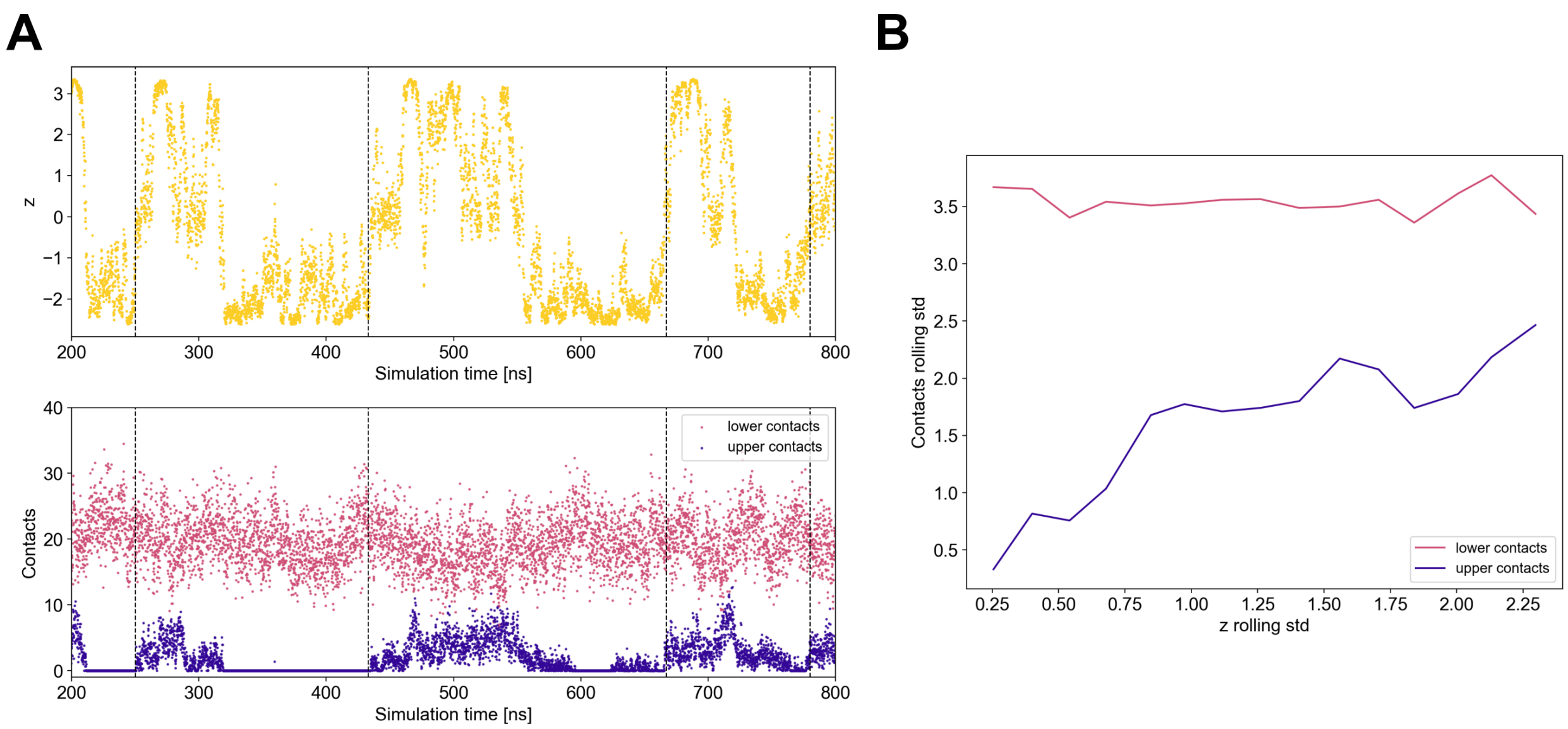

Figure S6: **Correlation between hydrophobic tail–ligand contacts and fluctuations of the committor variable. A:** Time evolution of the committor-related variable z (top, yellow) and of the number of contacts between lipid terminal tail beads and the hydrophobic beads of the NP ligands (bottom). Contacts are reported separately for lipids belonging to the lower membrane (pink) and upper membrane (purple). **B:** Rolling standard deviation of the lower- and upper-membrane contacts as a function of the rolling standard deviation of z. Fluctuations in z are accompanied by increasing fluctuations in upper-membrane contacts, whereas lower-membrane contacts remain constant. This suggests that rearrangements of the upper membrane are more closely associated with progress along the transition, while the lower membrane remains continuously perturbed by the embedded NP.

Supporting References

(1) Abraham, M. J.; Murtola, T.; Schulz, R.; Páll, S.; Smith, J. C.; Hess, B.; Lindahl, E. GROMACS: High Performance Molecular Simulations through Multi-Level Parallelism from Laptops to Supercomputers. *SoftwareX* **2015**, *1–2*, 19–25.

(2) Tribello, G. A.; Bonomi, M.; Branduardi, D.; Camilloni, C.; Bussi, G. PLUMED 2: New feathers for an old bird. *Computer physics communications* **2014**, *185*, 604–613.

(3) The PLUMED consortium. Promoting transparency and reproducibility in enhanced molecular simulations. *Nature Methods* **2019**, *16*, 670–673.

(4) Marrink, S. J.; Risselada, H. J.; Yefimov, S.; Tieleman, D. P.; de Vries, A. H. The MARTINI Force Field: Coarse Grained Model for Biomolecular Simulations. *The Journal of Physical Chemistry B* **2007**, *111*, 7812–7824.

(5) Bussi, G.; Donadio, D.; Parrinello, M. Canonical sampling through velocity rescaling. *The Journal of Chemical Physics* **2007**, *126*, 014101.

(6) Hub, J. S.; Awasthi, N. Probing a Continuous Polar Defect: A Reaction Coordinate for Pore Formation in Lipid Membranes. *Journal of Chemical Theory and Computation* **2017**, *13*, 2352–2366.

(7) Bartolo, A. L. D.; Masone, D. Synaptotagmin-1 C2B Domains Cooperatively Stabilize the Fusion Stalk via a Master-Servant Mechanism. *Chemical Science* **2022**, *13*, 3437–3446.
